# Derivation of a bronchial genomic classifier for lung cancer in a prospective study of patients undergoing diagnostic bronchoscopy

**DOI:** 10.1186/s12920-015-0091-3

**Published:** 2015-05-06

**Authors:** Duncan H Whitney, Michael R Elashoff, Kate Porta-Smith, Adam C Gower, Anil Vachani, J Scott Ferguson, Gerard A Silvestri, Jerome S Brody, Marc E Lenburg, Avrum Spira

**Affiliations:** Allegro Diagnostics, Corp, Maynard, MA USA; Elashoff Consulting, LLC, Redwood City, CA USA; Boston University School of Medicine, Boston, MA USA; University of Pennsylvania School of Medicine, Philadelphia, PA USA; University of Wisconsin School of Medicine and Public Health, Madison, WI USA; Medical University of South Carolina, Charleston, SC USA; Present affiliation: Veracyte, Inc, South San Francisco, CA USA

## Abstract

**Background:**

The gene expression profile of cytologically-normal bronchial airway epithelial cells has previously been shown to be altered in patients with lung cancer. Although bronchoscopy is often used for the diagnosis of lung cancer, its sensitivity is imperfect, especially for small and peripheral suspicious lesions. In this study, we derived a gene expression classifier from airway epithelial cells that detects the presence of cancer in current and former smokers undergoing bronchoscopy for suspect lung cancer and evaluated its sensitivity to detect lung cancer among patients from an independent cohort.

**Methods:**

We collected bronchial epithelial cells (BECs) from the mainstem bronchus of 299 current or former smokers (223 cancer-positive and 76 cancer-free subjects) undergoing bronchoscopy for suspected lung cancer in a prospective, multi-center study. RNA from these samples was run on gene expression microarrays for training a gene-expression classifier. A logistic regression model was built to predict cancer status, and the finalized classifier was validated in an independent cohort from a previous study.

**Results:**

We found 232 genes whose expression levels in the bronchial airway are associated with lung cancer. We then built a classifier based on the combination of 17 cancer genes, gene expression predictors of smoking status, smoking history, and gender, plus patient age. This classifier had a ROC curve AUC of 0.78 (95% CI, 0.70-0.86) in patients whose bronchoscopy did not lead to a diagnosis of lung cancer (n = 134). In the validation cohort, the classifier had a similar AUC of 0.81 (95% CI, 0.73-0.88) in this same subgroup (n = 118). The classifier performed similarly across a range of mass sizes, cancer histologies and stages. The negative predictive value was 94% (95% CI, 83-99%) in subjects with a non-diagnostic bronchoscopy.

**Conclusion:**

We developed a gene expression classifier measured in bronchial airway epithelial cells that is able to detect lung cancer in current and former smokers who have undergone bronchoscopy for suspicion of lung cancer. Due to the high NPV of the classifier, it could potentially inform clinical decisions regarding the need for further invasive testing in patients whose bronchoscopy is non diagnostic.

**Electronic supplementary material:**

The online version of this article (doi:10.1186/s12920-015-0091-3) contains supplementary material, which is available to authorized users.

## Background

Lung cancer remains the leading cause of cancer mortality in the United States, with an estimated 224,000 new diagnoses, and 160,000 deaths in 2014, 90% of which are due to smoking [1]. Recently, the National Lung Cancer Screening Trial showed that low dose Computed Tomography (CT) screening results in a 20% relative mortality reduction in high risk individuals [2]. The mortality reduction, however, was accompanied by a high rate (~96%) of false-positive CT findings, which in turn has generated concern for the overutilization of invasive diagnostic procedures [3].

Patients with suspected lung cancer are often referred for bronchoscopy where the primary aim is to sample a suspicious pulmonary lesion for pathological analysis. It is estimated that 500,000 bronchoscopies are performed per year in the U.S. [4], of which roughly half are for the diagnosis of lung cancer. Bronchoscopy is considered to be safer than other invasive sampling methods, such as transthoracic needle biopsy (TTNB), or surgical techniques. However the diagnostic sensitivity of bronchoscopy is sub-optimal, ranging from 34% (for <2 cm peripheral nodules) to 88% (for larger, centrally located lesions) [5]. Adoption of guidance techniques has expanded the applicability of bronchoscopy to more challenging suspicious lesions (i.e., solitary pulmonary nodules which are often peripheral in the lung), but the overall clinical sensitivity of bronchoscopy for lung cancer has not improved substantially [6,7]. When bronchoscopy is non-diagnostic, physicians are often left with the ambiguity of whether to pursue further invasive diagnostic procedures, with associated complications [8,9], or choose imaging surveillance. In current practice when these invasive procedures are performed, approximately a third of patients are determined to have benign disease [10,11], suggesting that these procedures are avoidable. Methods that reduce this ambiguity by substantially improving the diagnostic yield of bronchoscopy could improve patient care.

It has previously been demonstrated that cigarette smoke creates a molecular field of injury in airway epithelial cells that line the entire respiratory tract [12]. The reversible and irreversible impact of cigarette smoke on the bronchial airway transcriptome has been characterized and a set of gene-expression alterations in the bronchial epithelium have been identified in current and former smokers with lung cancer [13]. These cancer-associated gene expression profiles have previously been shown to yield a sensitive classifier for detecting lung cancer when bronchoscopy is non-diagnostic. The high sensitivity of this classifier, measured in a biospecimen readily accessible during bronchoscopy, results in a very low probability of lung cancer when the test result is negative, and suggests that physicians might be enabled to confidently pursue active surveillance and reduce risky invasive procedures in subjects without lung cancer.

We have expanded upon these proof of concept studies and conducted a prospective, multi-center study to derive a gene-expression classifier that could directly impact management of current and former smokers undergoing bronchoscopy for suspicion of lung cancer. We then validated the classifier in an independent cohort.

## Methods

### Training set patient population

Patients were enrolled in the AEGIS trials (Airway Epithelium Gene Expression In the DiagnosiS of Lung Cancer), designed as prospective, observational, cohort studies (registered as NCT01309087 and NCT00746759) of current and former cigarette smokers with a suspicion of lung cancer undergoing bronchoscopy as part of their diagnostic workup. A set of patients from one of the cohorts (“AEGIS 1”) was selected for the exclusive purpose of training a gene expression classifier. All enrolled patients were followed post-bronchoscopy until a final diagnosis was made, or for 12 months. Patients were diagnosed as having primary lung cancer based on cytopathology obtained at bronchoscopy or upon subsequent lung biopsy (such as TTNB or surgical lung biopsy (SLB) when bronchoscopy did not lead to a diagnosis of lung cancer). Patients were diagnosed as having benign disease based on a review of medical records and follow-up procedures at 12 months post-bronchoscopy (described in more detail in Additional file 1). Bronchoscopy was considered “diagnostic” when clinical samples collected at the time of the bronchoscopy procedure yielded a confirmed lung cancer diagnosis via cytology or pathology. The study was approved by IRB at each of the participating medical centers (the ethics committees and the study protocol numbers for each of the centers is listed separately; Additional file 2), and all patients signed an informed consent prior to enrollment.

### Sample collection

Physicians at each of 25 participating medical centers (see Additional file 3) were instructed to collect normal appearing bronchial epithelial cells (BEC) from the right mainstem bronchus (or the left side if any abnormalities were observed on the right) during bronchoscopy using standard bronchoscopic cytology brushes. Following collection, the cytology brushes were cut and placed in an RNA preservative (Qiagen RNAProtect, Cat. 76526) immediately after collection and stored at 4°C. Specimens were then shipped at 4-20°C to a central laboratory for further processing.

### RNA isolation

BECs were separated from cytology brushes using a vortex mixer and were then pelleted and processed using QIAzol lysis reagent (Qiagen). RNA was isolated by phenol/chloroform extractions and purified on a silica membrane spin-column (Qiagen miRNeasy kit, Cat. #217004) according to manufacturer’s recommendations. RNA was analyzed on a NanoDrop ND-1000 spectrophotometer (Thermo Scientific) to determine concentration and purity, and RNA integrity (RIN) was measured on a 2100 Bioanalyzer (Agilent Technologies). Each sample was then stored at −80°C until processing further on microarrays.

### Microarray processing

Total RNA (200 ng) was converted to sense strand cDNA, amplified using the Ambion WT Expression kit (Life Technologies Cat. #4440536), and labeled with Affymetrix GeneChip WT terminal labeling kit (Affymetrix Cat. #900671), (described in more detail in Additional file 1). The labeled cDNA was hybridized to Gene 1.0 ST microarrays (Affymetrix Cat. #901085) and analyzed on an Affymetrix GeneChip Scanner. Individual CEL files for each of the patient samples were normalized using the standard Affymetrix Gene 1.0 ST CDF and RMA [14].

### Classifier development

A gene expression classifier was derived in a multi-step process. Initial modeling consisted of using the training data to select genes which were associated with three clinical covariates (gender, tobacco use, and smoking history) to identify gene expression correlates of these clinical variables. Lung cancer-associated genes were then selected, and finally a classifier for predicting the likelihood of lung cancer based on the combination of the cancer genes, the gene expression correlates, and patient age was derived. All aspects of this classifier development procedure were determined using cross validation and using only data from the training set samples.

### Clinical Factor Gene Expression Correlates (CFGC)

Covariates of lung cancer in this study population, including sex (male/female), smoking status (current/former), and pack years (<10/>10), were modeled to identify gene expression correlates for the clinical factors. Empirical Bayes t-tests were used to identify genes whose expression was significantly associated with each of the clinical factors. Next, the significant genes were used to build three models, one for predicting each clinical factor, using penalized logistic regression (LASSO) [15]. Finally, the predicted values from the gene expression models for gender (GG), smoking status (GS), and pack-years (GPY) were computed, yielding genomic sex, genomic smoking status, and genomic pack year measures for each patient. These three genomic measures were used as new covariates to help in selecting genes with lung-cancer associated gene expression and in the lung cancer classifier (described below).

### Selection of lung cancer genes

A logistic regression model with lung cancer status (1 = cancer-positive and 0 = cancer-negative) as the dependent variable was fit using the training data, CFGC’s, and patient age as predictors. This model served as the “baseline” for subsequent gene expression analysis.

Next an empirical Bayes linear model was fit using gene expression values as the independent variable and the logistic regression baseline model residuals as the dependent variable. The residuals from this baseline model are a measure of patient cancer status that could not be predicted on the basis of clinical factors or their genomic correlates alone. That is, the empirical Bayes linear model was used to select genes with predictive potential for lung cancer independent or additive to that represented by clinical covariates. We note that a gene associated with both clinical factors and cancer could still be selected if the cancer association retained significance in this model. The top lung cancer-associated genes from this analysis were grouped using hierarchical clustering. To reduce the number of genes, for each cluster we selected a small number (2–4) of genes whose average was highly correlated to the average of all genes in the cluster. Subsequent modeling used these “reduced” cluster mean expression values rather than individual gene expression values. Cross validation was used to select which cluster means were independently significantly associated with lung cancer in the context of the other clusters. Overall, this served to select clusters that cumulatively provided the best classifier performance, and specific genes that best represented each of these clusters in a parsimonious manner. Functional analysis of genes within each of the cancer clusters was performed using DAVID [16] to identify biological terms describing the cancer-associated genes in the classifier.

### Lung cancer classifier

A lung cancer classifier was developed using lung cancer status as the outcome variable and a) the cancer associated gene expression cluster means, b) patient age, c) genomic gender (GG), d) genomic smoking status (GS), and e) genomic pack years (GPY) as predictors. The model was fit using a penalized logistic regression model; the penalization factor (lambda) was 0 for the clinical/ gene expression correlates and 10 for each of the gene expression cluster means. The resulting model score is on a 0 to 1 scale. A score threshold for predicting lung cancer status was established to achieve a sensitivity of approximately 90% for patients with a non-diagnostic bronchoscopy. An evaluation of the benefit of the gene expression classifier to predict lung cancer compared to clinical factors alone was performed by generating a “clinical model” that included age, gender, smoking status, and pack-years (all determined clinically) in a logistic regression model to predict lung cancer status. The difference in performance between the complete gene expression classifier and the clinical factors classifier to predict lung cancer status was assessed by comparing the AUC’s of each model in the training set.

### Analysis of an independent test set

Data from a prior study [13] were used as an independent test set to assess the performance of the locked classifier derived in this study. In that study BECs were collected at bronchoscopy from patients undergoing bronchoscopy for suspicion of lung cancer, and RNA was analyzed on microarrays (Affymetrix HG-U133A). CEL files from that study (n = 163) were re-normalized to produce gene-level expression values using Robust Multiarray Average (RMA) [14] in the Bioconductor R package (version 1.28.1). This processing used the Entrez Gene-specific probeset chip definition file (CDF) [17] in place of the standard U133A CDF provided by Affymetrix in order to facilitate cross-platform analyses. Analyses were performed using the R environment for statistical computing (version 2.9.2).

The classifier was applied to patients in the test set with two modifications to account for the difference in microarray platforms. First, the HG-U133A RMA expression values were adjusted by adding a gene-wise constant defined as the difference between the mean of the test set samples and the mean of the training set samples, separately for each gene. This procedure functioned to shift the mean of each gene’s expression levels in the test set to the mean observed in the training set. Second, for the classifier genes where a corresponding HG-U133A probeset was not available (LYPD2 and RNF150), the gene’s mean expression value in the training set was used for all of the test set samples.

### Statistical methods

Classifier accuracy was assessed using standard measures of prediction accuracy: the area under the curve (AUC), sensitivity, specificity, NPV and PPV. Cross-validation, using a 10% sample hold-out set, was used in the training set to estimate the performance of the prediction classifiers generated using these approaches [18]. These performance estimates were used to guide the development of the classifier discovery procedure. A final model was set prior to performing a one-time analysis of the test set. Fisher’s exact test was used to calculate statistical significance of all categorical variables (i.e., sex, smoking status, race, mass size, and mass location) and a t-test was used for continuous variables (i.e., age and smoking history).

## Results

### Study populations

A set of 299 patients from AEGIS 1 consisting of 223 patients diagnosed with lung cancer and 76 patients diagnosed with benign disease (Table 1) were used to derive our gene expression classifier. Characteristics of the independent test set have been previously described [13], and are summarized here (Additional file 4). Although the study design was similar to the one described here, there were some differences in the study populations. The patients were older on average in the training set compared to the test set (p < 0.001) (although there was no significant difference in age (p = 0.959) for patients diagnosed with lung cancer). The training set also consisted of fewer current smokers (p = 0.050); and a lower proportion of patients with < 3 cm lesions (p < 0.001). In addition, the prevalence of lung cancer was higher in the training set (75% versus 48%; p < 0.001).

**Table 1:** **Clinical and demographic characteristics of the patients used to train the classifier**

**Category**	**Sub-category**	**Lung cancer**	**Benign disease**	**p**
N		223	76	
Sex	Female	97	26	0.178
	Male	126	50	
Age (median years)		65	56	<0.001
Race	Caucasian	168	59	0.757
	African-American	47	13	
	Other	5	3	
	Unknown	3	1	
Smoking status	Current	101	26	0.107
	Former	122	50	
Smoking history (median PY)		43	30	<0.001
Mass size	<2 cm	46	23	<0.001
	>2 to <3 cm	30	12	
	≥3 cm	122	19	
	ill-defined infiltrate	10	13	
	Unknown	15	9	
Mass location	Central	86	16	0.018
	Peripheral	60	30	
	Central & peripheral	60	18	
	Unknown	17	12	
Histology	Sub-type			
SCLC		40		
NSCLC		180		
	Adenocarcinoma	83		
	Squamous	73		
	Large cell	6		
	Mixed/undefined	18		
Unknown		3		
Histology	Stage			
SCLC	Limited	16		
	Extensive	18		
	Unknown	6		
NSCLC	1	28		
	2	16		
	3	42		
	4	62		
	Unknown	32		
Benign disease	Sub-category			
Alternative diagnosis			54	
	Infection		23	
	Sarcoid		14	
	Inflammation		7	
	Fibrosis		4	
	Other		4	
	Benign growths		2	
Resolution/Stability			22	

The classifier training set included 223 patients diagnosed with lung cancer and 76 patients diagnosed with benign disease. The table lists clinical and demographic factors for all patients in the training set as well as characteristics of the lung cancer positive and patients with benign disease. The p-value for race is calculated for Caucasian versus non-caucasian.

### Derivation of the classifier and evaluation of performance

Gene expression was associated with current smoking status for a large fraction of the genes on the array (6477 genes with p < 0.001; top 10 genes reported in Additional file 5). Three of the top ranked genes (SLC7A11, TKT, and CLND10) were selected to serve as a logistic regression-based smoking status classifier based on cross-validation. This smoking status classifier had an AUC of 0.93 within the training set. An additional CFGC was derived for smoking history, independent of smoking status, and was based on cumulative smoke exposure, measured in pack-years. Smoking history (<10 PY vs > 10 PY) was significantly associated with the expression of 531 genes (p < 0.001; top 10 genes reported in Additional file 6). Two of the top genes were selected to serve as a logistic regression-based smoking history classifier (RUNX1T1, AKR1C2) which had an AUC of 0.78 within the training set. Sex was significantly associated with 339 genes (p < 0.001; top 10 genes reported in Additional file 7). The top ranked gene (RPS4Y1) was a perfect classifier (AUC = 1) of sex within the training set.

**Figure 1:**
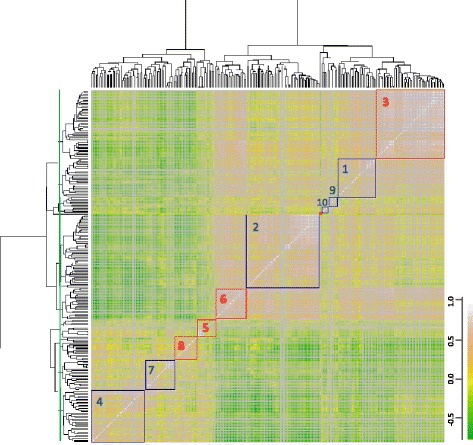
Pairwise correlation of genes with cancer-associated gene expression. The correlation between all possible pairs of genes with cancer-associated gene expression (n = 232) were assessed to identify groups of genes that share a similar pattern of gene expression. Unsupervised hierarchical clustering was used to group correlated genes into 11 clusters, with the dendrogram threshold level to establish clusters indicated on the y-axis (green line). Genes were selected from the clusters in a parsimonious manner to predict lung cancer status using linear regression. The classifier genes came from specific clusters (outlined in blue), using 2–4 genes from each cluster. Clusters 4 and 7 contain genes which were up-regulated in lung cancer, and clusters 1, 2, 9, and 10 were down-regulated in lung cancer.

As described in the methods, we identified genes whose expression is significantly associated with the residuals from the CFGC model for lung cancer. A total of 232 cancer associated genes (Additional file 8) met the significance criteria (T score > 2.7). A pairwise correlation of the 232 genes followed by hierarchical clustering was examined to identify genes with similar expression patterns and partitioned the genes into 11 clusters (Figure 1). Since genes were correlated within each cluster, we hypothesized that the mean of a small set of genes within each cluster could be used to represent the cluster in a sparse manner. We optimized the classifier, using cross validation to estimate the AUC. We selected genes to represent the gene clusters whose expression was most strongly associated with lung cancer and determined that inclusion of clusters 1, 2, 4, 7, 9 and 10 gave the best AUC. We also determined that beyond 2–4 genes per cluster the performance of the test did not improve. In cross-validation, AUC = 0.80 (95% CI 0.75 – 0.84) for all patients in the training set (n = 299); for the subset of patients with non-diagnostic bronchoscopy (n = 134) the performance was similar (AUC = 0.81; 95% CI 0.74 – 0.87).

The final lung cancer classifier was then determined using the finalized classifier discovery procedure on the entire training set. The classifier consisted of a combination of the six cancer gene clusters (represented by 17 genes in total), patient age, and the gene expression correlates (GG, GS, GPY) (Table 2) as predictors. Dichotomous classification was performed using a score threshold of 0.65 (patients with scores >/= 0.65 were predicted as cancer-positive and <0.65, cancer-negative). The classifier had a sensitivity of 93% and specificity of 57% in the training set and there was no difference in the AUC of the classifier for the entire training set (0.78; 95% CI, 0.73-0.82), compared with the subset of patients whose bronchoscopy was non-diagnostic for lung cancer (AUC = 0.78; 95% 0.71-0.85), (see, Additional file 9). We also found that there was no difference in the AUC (p = 0.62) comparing Caucasians and African-Americans (the two predominant races in the training set), although the former smoked significantly more (p = 0.03), with a mean PY difference of 46 versus 38, respectively (Additional file 10).

**Table 2:** **Description of the gene expression classifier** ^**a**^

**Feature** ^**b**^ **, (x**_**i**_ **)**	**Coefficient, (b**_**i**_ **)**	**Genes within features**			
Age	0.0623				
GG	0.5450	RPS4Y1			
GS	0.1661	SLC7A11	CLND10	TKT	
GPY	3.0205	RUNX1T1	AKR1C2		
CA (1)	−0.4406	BST1	CD177.1	CD177.2	
CA (2)	−0.3402	ATP12A	TSPAN2		
CA (4)	0.1725	GABBR1	MCAM	NOVA1	SDC2
CA (7)	0.5670	CDR1	CGREF1	CLND22	NKX3-1
CA (9)	−0.3160	EPHX3	LYPD2		
CA (10)	−0.3791	MIA	RNF150		
Intercept (b_0_)	3.3173				

^a)^Genomic gender was defined as GG = 1 (female) if RPS4Y1 < 7.5, 0 (male) otherwise. The predicted genomic smoking (GS) value was derived, where x = 40.8579-0.4462*SLC7A11-2.1298*CLND10-1.8256*TKT, and genomic smoking GS = e^*x*^/(1+ e^*x*^). The predicted genomic pack years (GPY) value was derived, where x = −5.1429 + 2.1891*RUNX1T1 -0.9506*AKR1C2, and genomic pack years GPY = exp(x)/(1 + exp(x)). The generalized equation for the prediction classifier was: Score = e^*y*^/(1+ e^*y*^), where, y = b_*0*_ + Ʃ(b_i_*x_*i*_), where b_*0*_ is the intercept, b_*i*_ is the coefficient, and x_*i*_ is the feature (as shown).

^b)^Features include patient age (as reported), GG, GS, GPY as described in the methods, and CA (*i*), the lung cancer gene clusters (shown in Figure 1).

The gene expression classifier performed significantly better (AUC = 0.78; 95% CI, 0.73-0.82) than a model using clinical factors alone (AUC = 0.72; 95% CI, 0.67-0.77) in the training set (p < 0.001). Functional analysis of the 17 cancer genes is summarized separately (Additional file 11). Nine of the genes are down-regulated and eight are up-regulated in association with cancer.

### Validation in an independent test set

**Figure 2:**
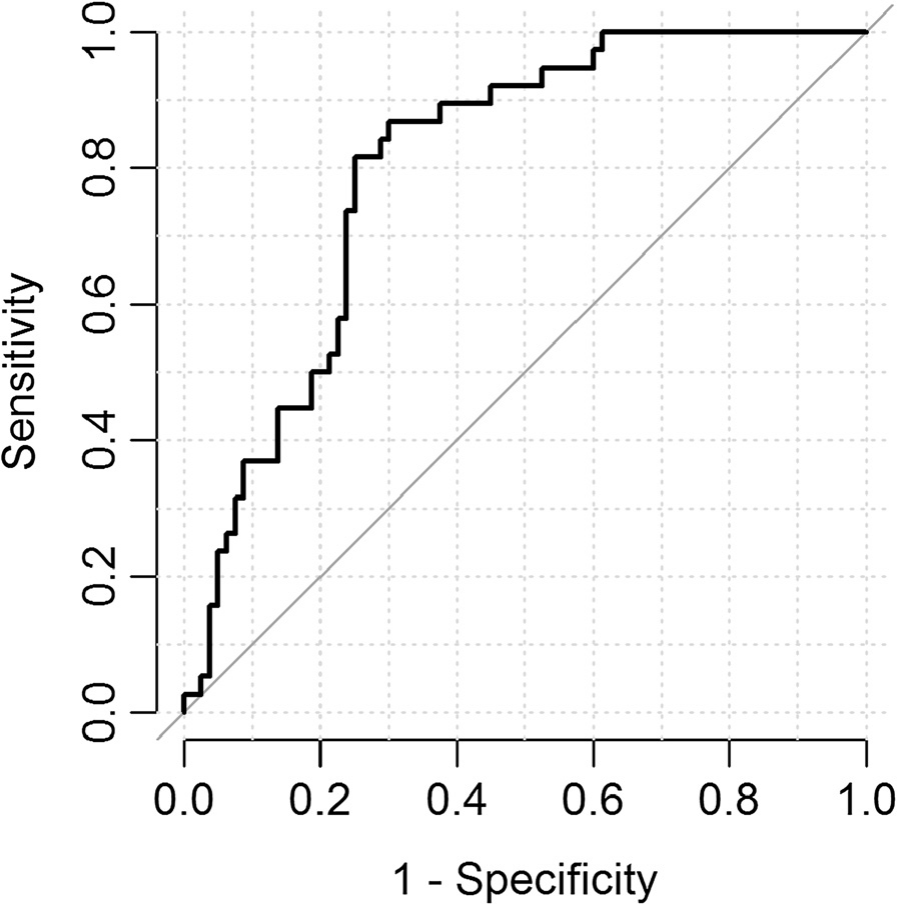
ROC curve of patients with a non-diagnostic bronchoscopy in the test set. The AUC = 0.81 for the 123 patients whose bronchoscopy did not result in a diagnosis of lung cancer (in which the prevalence of lung cancer = 31%).

In the patients with non-diagnostic bronchoscopy (n = 123) of the independent test set, the AUC of the classifier was 0.81 (95% CI, 0.73 – 0.88), (Figure 2) which was similar to the performance in patients with non-diagnostic bronchoscopy in the training set (AUC = 0.78; 95% 0.71-0.85; p = 0.495). The sensitivity was 92% and with a specificity of 53%, the NPV was 94% (95% CI, 83-99%), (see Table 3). Interestingly we did not observe any effect of cancer histology, cancer stage (Table 4), or lesion size (Table 5) on the classifier’s sensitivity for cancer. Moreover, in the test set the classifier had an AUC of 0.79 in current smokers and 0.82 in former smokers, suggesting that smoking status does not have a significant effect on classifier performance (p = 0.710). When compared with bronchoscopy alone, the combination of the gene expression classifier with bronchoscopy improved the sensitivity from 51% to 96% (p <0.001).

**Table 3:** **Performance of bronchoscopy, classifier, and the combined procedures in the test set**

**Category**	**Bronchoscopy**	**Classifier** ^*^	**Classifier & bronchoscopy combined**
N, total	163	123	163
N, Lung cancer	78	38	78
N, Benign disease	85	85	85
Sensitivity (95% CI)	51% (40-62%)	92% (78-98%)	96% (89-99%)
Specificity (95% CI)	100% (95-100%)	53% (42-63%)	53% (42-63%)
NPV (95% CI)	69% (60-77%)	94% (83-98%)	94% (83-98%)
PPV (95% CI)	100% (90-100%)	47% (36-58%)	65% (56-73%)

*The performance of the classifier was evaluated in patients in which bronchoscopy did not result in a finding of cancer (n = 123).

**Table 4:** **Sensitivity of bronchoscopy, the classifier, and the combined procedures for patients with lung cancer in the test set**

**Histology**	**Sub-type**	**N**	**Bronchoscopy sensitivity**	**Classifier sensitivity**	**Combined sensitivity**
All cancers		78	51%^a^	92%^b^	96%^c^
SCLC		14	64%	100%	100%
NSCLC		64	48%	91%	95%
	Adenocarcinoma	18	33%	83%	89%
	Squamous	27	56%	92%	96%
	Large cell	4	25%	100%	100%
	Undefined	15	60%	83%	93%
Histology	Stage				
SCLC					
	Limited	9	78%	100%	100%
	Extensive	5	40%	100%	100%
NSCLC					
	1	14	36%	100%	100%
	2	2	50%	100%	100%
	3	25	52%	92%	96%
	4	22	55%	80%	91%
	Unknown	1	0%	100%	100%

Of 163 patients who underwent a diagnostic bronchoscopy procedure for suspicion of lung cancer, 78 were diagnosed with cancer. A lung cancer diagnosis was made at bronchoscopy (a) in 40 patients (51%; 95% CI, 40-62%), and in the remaining lung cancer patients where no diagnosis was made at bronchoscopy, (b) the classifier correctly predicted 34 of them (89%; 95% CI, 75-96%). The classifier combined with bronchoscopy (c) yielded a detection of 74 of 78 (95%; 95% CI, 87-98%) patients with lung cancer. The sensitivities of bronchoscopy, the classifier, and the combined procedures are also shown for lung cancers according to sub-type and stage.

**Table 5:** **Sensitivity of bronchoscopy, the classifier, and the combined procedures in the test set stratified by size of suspicious lesions**

Mass size*	**N**	**Bronchoscopy sensitivity**	**Classifier sensitivity**	**Combined sensitivity**
<3 cm	99	44%	87%	93%
>3 cm	48	58%	94%	98%
Ill-def Infiltrate	16	38%	100%	100%

*Includes patients diagnosed with lung cancer and those with benign disease.

## Discussion

Previous work has demonstrated that there are persistent gene-expression alterations in normal epithelial cells from the bronchial airway that are associated with exposure to cigarette smoke and the presence of lung cancer in current and former smokers [12,19-21]. These cancer-associated differences can be used to derive classifiers capable of accurately detecting lung cancer in these relatively non-invasively collected biospecimens obtained during bronchoscopy [13]. In current practice it is challenging to rule out lung cancer when bronchoscopy does not lead to a finding of malignancy, and the false-negative rate can range from 20-70% [5]. Current guidelines suggest that patients with elevated risk of disease should be pursued with more invasive follow-up diagnostic procedures [5], which carry increased risk of complications [8]. However due to uncertainty these procedures often performed in patients found to have benign disease [10,11]. Therefore our goal was to derive a gene-expression classifier using epithelial cells collected from the normal-appearing proximal airway during bronchoscopy that could be used in combination with bronchoscopy to increase the overall sensitivity and negative predictive value for lung cancer diagnosis. A classifier with high sensitivity and high NPV among current or former smokers with a non-diagnostic bronchoscopy could serve to significantly reduce the probability of lung cancer in this clinical setting, reducing the use of additional unnecessary invasive procedures in smokers with benign lung lesions.

In this study, we leveraged a cohort of current and former smokers undergoing bronchoscopy for suspected lung cancer from a larger multicenter study to derive a gene-expression classifier for lung cancer. The classifier is a multivariate logistic regression model that has high sensitivity and high NPV. Importantly, we have validated the performance of the classifier in an independent cohort, using data from a previously published study of airway samples collected from smokers undergoing bronchoscopy for suspected lung cancer. The sensitivity is 92% in patients whose bronchoscopy is non-diagnostic in the test set with a specificity of 53%. The NPV is 94% in the test set compared to an NPV of 69% for bronchoscopy alone suggesting that the classifier could help physicians reliably identify patients unlikely to have lung cancer after a non-diagnostic bronchoscopy. Given the different microarray platform (Affymetrix HU133A) used in the test set, the analysis was done with a classifier lacking LYPD2 and RNF150 since these genes were not measured on that microarray platform. However, the two genes added to classifier performance during cross-validation in the training data and the full classifier has subsequently been validated in two additional datasets (manuscript in preparation).

The functions of the differentially expressed genes in the normal appearing airway epithelium in current and former smokers with lung cancer provide insight into the biology underlying the field of injury (see Additional file 8). Among genes that are suppressed, there are a number involved in the immune response, including CD177 and BST1, suggesting an impaired immune response in the airway of smokers with lung cancer. The gene TSPAN2, whose expression is depressed by p53 knockdown and is associated with poor prognosis in lung adenocarcinomas [22], was also expressed at lower levels in patients with cancer. Also EPHX3, a gene involved in xenobiotic metabolism, processing of carcinogens in tobacco smoke, and carcinogenesis in other epithelial cancers is down-regulated [23]. Among the classifier genes that are up-regulated in lung cancer, NOVA1 and CDR1 are predominantly expressed in neurons, but are also expressed in tumors and are associated with para-neoplastic antibodies in several malignancies, including small-cell lung cancer [24-28]. Furthermore, MCAM which is up-regulated in lung cancer, is expressed in basal bronchial epithelial cells [29]. MCAM is also strongly and transiently up-regulated in tracheal epithelium during repair [30], is required for tracheal epithelial regeneration [31], and is up-regulated in the bronchial epithelium of patients with COPD [32] and asthma [33]. A number of classifier genes that regulate cell growth and proliferation are up-regulated in patients with lung cancer, including SDC2, and NKX3-1 as well as the cell-cycle-arrest mediator CGREF1. Finally the CFGC genes selected to predict smoking status (SLC7A11, CLDN10, TKT) and smoking history (RUNX1T1, AKR1C2) in our classifier have been previously reported as being altered by tobacco smoke exposure, confirming the robust effect of smoking on airway epithelium biology [12,19,34].

Our discovery approach extends earlier work on gene-expression based lung cancer diagnostics [13] primarily in the explicit modeling of clinical covariates as components of the predictive model prior to selection of features with lung cancer-associated expression. It is known that the response to environmental insults and other clinical factors can vary substantially between individuals. Therefore our approach was to use gene expression to capture the patient-level physiological response to an environmental insult (e.g., cumulative smoke exposure), as this response may be more reflective of disease risk than the actual reported values [35]. Additionally, the use of gene expression data to predict critical data inputs (such as patient sex) minimizes potential for data entry errors in clinical practice. Another component of our approach was selecting genes whose expression is associated with cancer after accounting for the modeled clinical factors. We hypothesized that this approach would help ensure that the information about the likelihood of cancer captured by the genes with cancer-associated gene expression is independent from the information about cancer captured by the modeled clinical factors. An additional important aspect of our classifier discovery approach was our methodology to identify patterns of independent cancer-associated gene expression through clustering and then to model cancer as the additive effects of each of the cancer-associated gene expression modules. This is in contrast to selecting only genes that are globally top-ranked according to their association with cancer which could potentially result in selecting an entire panel of genes that reflect a single cancer-associated molecular process. Previous studies to derive a gene expression classifier to predict risk of lung cancer in normal appearing airway epithelial cells have described similar results with high sensitivity and NPV when bronchoscopy is non-diagnostic [13]. While there are no common genes in that classifier compared to the one described here, we believe that our new classifier represents similar mechanisms of action given the strong performance in the independent test set. However, the differences in the specific genes selected in the classifier described here may be due to differences in the feature selection process, specifically, the method of accounting for gene expression strongly associated with clinical covariates while selecting cancer genes.

## Conclusion

We have derived a gene expression classifier for lung cancer in current and former smokers using cells from the proximal airway that can be used in conjunction with bronchoscopy for suspected lung cancer. We have validated the performance of this classifier in an independent test set. The classifier adds substantial sensitivity to the bronchoscopy procedure resulting in high NPV. This classifier can be used to aid in decision-making when bronchoscopy is non-diagnostic by identifying patients who are at low risk of having lung cancer.

## Additional files

Additional file 1:
**Supplemental Methods.**
Additional file 2:
**Ethics committees of the AEGIS 1 study.**
Additional file 3:
**Medical centers in which the AEGIS 1 clinical cohort was enrolled.**
Additional file 4:
**Demographic and clinical characteristics of the test set patients.**
Additional file 5:
**Top differentially expressed genes associated with smoking status.**
Additional file 6:
**Top differentially expressed genes associated with smoking history.**
Additional file 7:
**Top differentially expressed genes associated with gender.**
Additional file 8:
**Genes associated with cancer which are included in the classifier.**
Additional file 9:
**ROC curve analysis of the training set cohort using the finalized gene expression classifier.**
Additional file 10:
**Analysis of clinical characteristics and classifier performance according to race.**
Additional file 11:
**Biological characterization of classifier genes.**
