# Supplementary material for: Derivation of a bronchial genomic classifier for lung cancer in a prospective study of patients undergoing diagnostic bronchoscopy
Source: BMC Med Genomics. 2015 May 6;8:18. doi: 10.1186/s12920-015-0091-3 (PMC4434538; doi:10.1186/s12920-015-0091-3)
Supplement: Additional file 1: — Supplemental Methods. [file 12920_2015_91_MOESM1_ESM.docx]

**Additional File 1:** Supplemental Methods

## Study Population

Patients enrolled in the NIH-registered AEGIS studies (NCT01309087 and NCT00746759) were patients undergoing clinically indicated bronchoscopy for suspicion of lung cancer who were at least 21 years of age and had smoked at least 100 cigarettes in their lifetime. Study exclusions included patients who had previously been diagnosed with primary lung cancer, who had been on a mechanical ventilator for ≥ 24 consecutive hours immediately prior to bronchoscopy, or who could not consent or comply with the study.  Additional patients were excluded prior to training the classifier to exclude patients with a malignancy other than primary lung cancer. This included the exclusion of patients with a history of any malignancy, confirmed metastatic cancer to the lung, or found to have an active non-lung primary cancer after enrollment. Also, patients without a final definitive diagnosis were excluded. Finally, after specimen processing, those with insufficient yield (<1µg) or quality (RIN<4) of RNA were excluded from further analysis.

Patients were followed for up to twelve months post bronchoscopy and records were reviewed to confirm or determine a final clinical diagnosis. A diagnosis of cancer was based on cytopathology of cells/tissue collected either during bronchoscopy, or in follow-up procedures when bronchoscopy was non-diagnostic. Follow-up procedures leading to diagnosis consisted of a second bronchoscopy, transthoracic needle aspiration (TTNA), surgical lung biopsy (SLB), or a combination of procedures. Records of patients who were not diagnosed with cancer, and who had been followed for 12 months, underwent an adjudication process by a panel of five pulmonologists. The process consisted of a review of the available medical records and patients were only declared to be cancer-free if the patient met one of the following criteria: diagnosed with an alternative diagnosis that explained the initial suspicious abnormality, the abnormality was determined to be stable, or the abnormality resolved. Patients who did not meet these criteria at the completion of the 12-month follow-up period were labeled as “indeterminate” and were excluded from training, due to lack of diagnostic “truth”.

## Sample Collection

A shipping container was provided to all sites enabling the transport of specimens at 4-20⁰C within a 48 hour period. Sites were asked to send specimens using 2-day shipping services. Upon receipt in the central laboratory, specimens were inspected and accessioned into a laboratory information system. Accepted specimens were stored at 4⁰C prior to RNA isolation, which was typically conducted within 7 days of receipt. Records of all storage, and shipping times were retained, and the cumulative time between specimen collection and RNA isolation was less than 30 days (consistent with manufacturer’s recommendations for the RNA preservative).

## Microarray Processing

Total RNA was converted to sense-strand cDNA using a commercial kit (Ambion WT; Life Technologies, Cat. # 4440536) designed for use with Affymetrix microarrays. Starting with 200ng of total RNA, single stranded cDNA was prepared through reverse transcription using T7 promoter primers protocol. Single-strand cDNA was converted to double stranded cDNA using DNA polymerase. Double stranded cDNA acts as a template for *in vitro* transcription of cRNA which was then purified to remove enzymes, salts, inorganic phosphates and unincorporated nucleotides. The yield of cRNA was measured using UV-adsorption and labeled sense-stranded cDNA was then generated using 10 µg of the purified cRNA by reverse transcription with random primers and a mix of dUTP/dNTPs, fragmented, and labeled using the GeneChip WT Terminal labeling kit (Affymetrix, Cat. #900671).  The labeled cDNA was hybridized to Gene 1.0 ST microarrays (Affymetrix Cat. #901085) using the Hybridization, Wash and Stain kit (Affymetrix Cat. #900720), and incubated at 45°C for 16 hours. Following hybridization, arrays were washed and stained using standard Affymetrix procedures before being scanned on the Affymetrix GeneChip Scanner, and data was extracted using Expression Console software (Affymetrix). 
