# Supplementary material for: Derivation of a bronchial genomic classifier for lung cancer in a prospective study of patients undergoing diagnostic bronchoscopy
Source: BMC Med Genomics. 2015 May 6;8:18. doi: 10.1186/s12920-015-0091-3 (PMC4434538; doi:10.1186/s12920-015-0091-3)
Supplement: Additional file 2: — Ethics committees of the AEGIS 1 study. [file 12920_2015_91_MOESM2_ESM.docx]

**Additional file 2** – Ethics committees of the AEGIS 1 study

| Medical Center | Ethics Committee Name | Protocol # |
| --- | --- | --- |
| University of Pennsylvania | U. Pennsylvania Regulatory Affairs | 809172 |
| Louisiana State University | LSU Health Sciences Center | 7199 |
| Georgia Lung Associates | WIRB | 20090312 |
| Medical University of South Carolina | MUSC IRB | 20008 |
| Beth Israel Deaconess Medical Center | BIDMC Committee on Clinical Investigations | 2008P-000406 |
| University of Missouri – Columbia | Health Sciences Institutional Review Board | 1128277 |
| Trinity College | Joint Research Ethics Committee | 031201/19303 |
| Temple University | TU Human Subjects Protections IRB | 12231 |
| North Florida/South Georgia Veterans | UF Health center Institutional Review Board | 633-2010 |
| Virginia Commonwealth University | Western Institutional Review Board | 20090312 |
| William Jennings Bryan Dorn Veterans | WJBD VA Medical Center IRB | 10305 |
| Vanderbilt University | VU IRB | IRB# 081239 |
| New York University | NYU School of Medicine IRB | H08-803 |
| University of Virginia-Charlottesville | UVA IRB for Health Sciences Research | HSR #14315 |
| Columbia University | Columbia University Medical Center IRB | AAAC: 971 |
| Indiana University | IU Human Subjects Office | 1105005635/0902-02 |
| Pulmonary and Allergy Associates, P.A. | Western Institutional Review Board | 20090312 |
| University of Wisconsin - Madison | Western Institutional Review Board | 20090312 |
| National Jewish Health | Western Institutional Review Board | 20090312 |
| St. Elizabeth's Medical Center | St. Elizabeth's Medical Center IRB | 554 |
| University of Alabama - Birmingham | WIRB | 1106381 |
| Pulmonary Associates | WIRB | 20090312 |
| University of British Columbia | UBC Cancer Agency Research Ethics Board | H08-02354 |
| Overlake Hospital | WIRB | 20090312 |
| Jamaica Hospital Medical Center | Jamaica Hospital Medical Center IRB | 244625 |
