# Supplementary material for: Derivation of a bronchial genomic classifier for lung cancer in a prospective study of patients undergoing diagnostic bronchoscopy
Source: BMC Med Genomics. 2015 May 6;8:18. doi: 10.1186/s12920-015-0091-3 (PMC4434538; doi:10.1186/s12920-015-0091-3)
Supplement: Additional file 3: — Medical centers in which the AEGIS 1 clinical cohort was enrolled. [file 12920_2015_91_MOESM3_ESM.docx]

**Additional file 3**: Medical centers in which the AEGIS 1 clinical cohort was enrolled

| Principle Investigator | Medical Center | Site Location | Site Type |
| --- | --- | --- | --- |
| A. Vachani | University of Pennsylvania | Philadelphia, PA | Academic |
| S. Kantrow | Louisiana State University | New Orleans, LA | Academic |
| S. Simon | Georgia Lung Associates | Austell, GA | Community |
| G. Silvestri | Medical University of South Carolina | Charleston, SC | Academic |
| A. Ernst & G. Michaud | Beth Israel Deaconess Medical Center | Boston, MA | Academic |
| V. Guntur | University of Missouri – Columbia | Columbia, MO | Academic |
| J. Keane | Trinity College | Dublin, IRE | Academic |
| J. Travaline | Temple University | Philadelphia, PA | Academic |
| P. Sriram | North Florida/South Georgia Veterans | Gainesville, FL | Military |
| R. Shepherd | Virginia Commonwealth University | Richmond, VA | Academic |
| B. Smith & A. Mass | William Jennings Bryan Dorn Veterans | Columbia, SC | Military |
| P.Massion | Vanderbilt University | Nashville, TN | Academic |
| W. Rom & E. Leibert | New York University | New York, NY | Academic |
| G. Verghese | University of Virginia-Charlottesville | Charlottesville, VA | Academic |
| C. Powell | Columbia University | New York, NY | Academic |
| F. Sheski | Indiana University | Indianapolis, IN | Academic |
| R. Sussman | Pulmonary and Allergy Associates, P.A. | Summit, NJ | Community |
| J.S. Ferguson | University of Wisconsin - Madison | Madison, WI | Academic |
| A. Musani | National Jewish Health | Denver, CO | Academic |
| A. Ernst & S. Rafeq | St. Elizabeth's Medical Center | Brighton, MA | Community |
| M. Dransfield | University of Alabama - Birmingham | Birmingham, AL | Academic |
| M. Gotfried | Pulmonary Associates | Phoenix, AZ | Community |
| S. Lam | University of British Columbia | British Columbia, CAN | Academic |
| A. Markezich | Overlake Hospital | Bellevue, WA | Community |
| C. Thurm | Jamaica Hospital Medical Center | Jamaica, NY | Community |
