# Supplementary material for: Derivation of a bronchial genomic classifier for lung cancer in a prospective study of patients undergoing diagnostic bronchoscopy
Source: BMC Med Genomics. 2015 May 6;8:18. doi: 10.1186/s12920-015-0091-3 (PMC4434538; doi:10.1186/s12920-015-0091-3)
Supplement: Additional file 4: — Demographic and clinical characteristics of the test set patients. [file 12920_2015_91_MOESM4_ESM.docx]

**Additional file 4:** Demographic and clinical characteristics of the test set patients

| Category | Sub-category | Benign  Disease | Lung  Cancer | Total |
| --- | --- | --- | --- | --- |
| N |  | 85 | 78 | 163 |
| Gender | Female | 23 | 18 | 41 |
|  | Male | 62 | 60 | 122 |
| Median Age |  | 52 | 65 |  |
| Smoking status | Current | 51 | 35 | 86 |
|  | Former | 34 | 43 | 77 |
| Smoking History (PY) |  | 36 | 55 |  |
| Lesion size (cm) | <2 | 68 | 18 | 86 |
|  | 2-3 | 4 | 9 | 13 |
|  | >3 | 3 | 45 | 48 |
|  | Infiltrate | 8 | 8 | 16 |
| Histology | Sub-type | Stage |  |  |
| SCLC |  |  | 14 |  |
|  |  | Limited | 9 |  |
|  |  | Extensive | 5 |  |
| NSCLC |  |  | 64 |  |
|  | Adenocarcinoma |  | 18 |  |
|  | Squamous |  | 27 |  |
|  | Large cell |  | 4 |  |
|  | Unspecified |  | 15 |  |
|  |  | 1 | 14 |  |
|  |  | 2 | 2 |  |
|  |  | 3 | 25 |  |
|  |  | 4 | 22 |  |
|  |  | Unknown | 1 |  |

Former smokers are defined as quitting more than 30 days prior to the scheduled bronchoscopy and smoking history is reported as median pack-years (PY).
