# Supplementary material for: Derivation of a bronchial genomic classifier for lung cancer in a prospective study of patients undergoing diagnostic bronchoscopy
Source: BMC Med Genomics. 2015 May 6;8:18. doi: 10.1186/s12920-015-0091-3 (PMC4434538; doi:10.1186/s12920-015-0091-3)
Supplement: Additional file 5: — Top differentially expressed genes associated with smoking status. [file 12920_2015_91_MOESM5_ESM.docx]

**Additional File 5:** Top differentially expressed genes associated with smoking status

| ID | Symbol | logFC | AveExpr | T | P.Value | GS term |
| --- | --- | --- | --- | --- | --- | --- |
| 8102800 | SLC7A11 | -2.31513 | 7.80246 | -19.0302 | 1.44E-53 | YES |
| 8088106 | TKT | -0.70341 | 9.137779 | -18.3095 | 7.46E-51 | YES |
| 8084630 | NA | -1.39188 | 6.897858 | -18.2898 | 8.86E-51 |  |
| 8136336 | AKR1B10 | -2.27454 | 6.839364 | -18.2346 | 1.43E-50 |  |
| 7969640 | CLDN10 | -0.94118 | 9.579322 | -18.1835 | 2.23E-50 | YES |
| 8171435 | PIR | -0.80298 | 8.924225 | -18.0114 | 9.97E-50 |  |
| 7937465 | TALDO1 | -0.62614 | 10.07378 | -17.8271 | 4.95E-49 |  |
| 8051583 | CYP1B1 | -2.8955 | 8.179293 | -17.7765 | 7.69E-49 |  |
| 8020653 | CABYR | -1.19744 | 8.009791 | -17.6885 | 1.65E-48 |  |
| 7979658 | GPX2 | -1.10719 | 10.62466 | -17.524 | 6.93E-48 |  |
