# Supplementary material for: Derivation of a bronchial genomic classifier for lung cancer in a prospective study of patients undergoing diagnostic bronchoscopy
Source: BMC Med Genomics. 2015 May 6;8:18. doi: 10.1186/s12920-015-0091-3 (PMC4434538; doi:10.1186/s12920-015-0091-3)
Supplement: Additional file 6: — Top differentially expressed genes associated with smoking history. [file 12920_2015_91_MOESM6_ESM.docx]

**Additional file 6:** Top differentially expressed genes associated with smoking history

| ID | Symbol | logFC | AveExpr | T | P.Value | GPY term |
| --- | --- | --- | --- | --- | --- | --- |
| 8151768 | RUNX1T1 | 0.435653 | 5.905711 | 8.547091 | 6.44E-16 | Yes |
| 8077989 | TPRXL | -0.36913 | 8.733733 | -6.22283 | 1.63E-09 |  |
| 7994058 | SCNN1G | 0.685624 | 8.450023 | 5.90033 | 9.75E-09 |  |
| 8069764 | NA | -0.32511 | 7.23309 | -5.82162 | 1.49E-08 |  |
| 8145470 | DPYSL2 | 0.260084 | 7.62917 | 5.749689 | 2.19E-08 |  |
| 7931832 | AKR1C2 | -0.81612 | 10.93402 | -5.72526 | 2.50E-08 | Yes |
| 8039674 | ZNF154 | 0.372911 | 7.366637 | 5.724589 | 2.51E-08 |  |
| 8150978 | CA8 | 0.415392 | 6.182963 | 5.638727 | 3.95E-08 |  |
| 8129497 | EPB41L2 | 0.4248 | 6.895569 | 5.589681 | 5.10E-08 |  |
| 8039672 | NA | 0.437196 | 4.729487 | 5.515997 | 7.48E-08 |  |
