# Supplementary material for: Derivation of a bronchial genomic classifier for lung cancer in a prospective study of patients undergoing diagnostic bronchoscopy
Source: BMC Med Genomics. 2015 May 6;8:18. doi: 10.1186/s12920-015-0091-3 (PMC4434538; doi:10.1186/s12920-015-0091-3)
Supplement: Additional file 7: — Top differentially expressed genes associated with gender. [file 12920_2015_91_MOESM7_ESM.docx]

**Additional file 7:** Top differentially expressed genes associated with gender

| ID | Symbol | logFC | AveExpr | T | P.Value | GS term |
| --- | --- | --- | --- | --- | --- | --- |
| 8176375 | RPS4Y1 | -3.16276 | 7.851579 | -84.6047 | 6.46E-212 | YES |
| 8176624 | DDX3Y | -4.05255 | 7.702569 | -84.0882 | 3.79E-211 |  |
| 8177232 | KDM5D | -2.23316 | 7.443775 | -80.4804 | 1.17E-205 |  |
| 8176578 | USP9Y | -3.35997 | 7.485794 | -79.3437 | 7.01E-204 |  |
| 8177137 | UTY | -3.38728 | 7.655898 | -78.9665 | 2.76E-203 |  |
| 8176698 | TXLNG2P | -2.82024 | 6.904035 | -70.7168 | 1.36E-189 |  |
| 8176709 | CYorf15B | -2.63878 | 7.07696 | -68.9368 | 1.87E-186 |  |
| 8176719 | EIF1AY | -3.02926 | 7.079489 | -66.6741 | 2.34E-182 |  |
| 8176384 | ZFY | -1.6795 | 6.679083 | -59.2385 | 5.47E-168 |  |
| 8176460 | PRKY | -1.33643 | 7.761388 | -52.0418 | 1.48E-152 |  |
