# Supplementary material for: Derivation of a bronchial genomic classifier for lung cancer in a prospective study of patients undergoing diagnostic bronchoscopy
Source: BMC Med Genomics. 2015 May 6;8:18. doi: 10.1186/s12920-015-0091-3 (PMC4434538; doi:10.1186/s12920-015-0091-3)
Supplement: Additional file 8: — Genes associated with cancer which are included in the classifier. [file 12920_2015_91_MOESM8_ESM.docx]

**Additional file 8:** Genes associated with cancer which are included in the classifier

| ID | Symbol | T | p.value | FC | Cluster | Final Model |
| --- | --- | --- | --- | --- | --- | --- |
| 8094228 | BST1 | -4.29031 | 2.41E-05 | 0.89208 | 1 | Yes |
| 8037298 | CD177 | -3.85704 | 0.00014 | 0.715357 | 1 | Yes |
| 8029280 | CD177 | -3.68455 | 0.000272 | 0.840725 | 1 | Yes |
| 7918857 | TSPAN2 | -3.92967 | 0.000106 | 0.845904 | 2 | Yes |
| 7968062 | ATP12A | -3.49107 | 0.000553 | 0.794623 | 2 | Yes |
| 8124654 | GABBR1 | 2.881256 | 0.004247 | 1.071879 | 4 | Yes |
| 8147461 | SDC2 | 2.847433 | 0.004712 | 1.089453 | 4 | Yes |
| 7978391 | NOVA1 | 2.729315 | 0.006721 | 1.094912 | 4 | Yes |
| 7952205 | MCAM | 2.70666 | 0.007186 | 1.06072 | 4 | Yes |
| 8175531 | CDR1 | 4.307308 | 2.24E-05 | 1.468199 | 7 | Yes |
| 8103877 | CLDN22 | 3.502336 | 0.000531 | 1.329189 | 7 | Yes |
| 8051001 | CGREF1 | 3.275505 | 0.001178 | 1.056672 | 7 | Yes |
| 8149811 | NKX3-1 | 2.92659 | 0.003689 | 1.175825 | 7 | Yes |
| 8034974 | EPHX3 | -3.73504 | 0.000225 | 0.898923 | 9 | Yes |
| 8153342 | LYPD2 | -2.93177 | 0.00363 | 0.887468 | 9 | Yes |
| 8102938 | RNF150 | -4.32839 | 2.05E-05 | 0.880745 | 10 | Yes |
| 8028924 | MIA | -3.23844 | 0.001337 | 0.906368 | 10 | Yes |
