# Supplementary material for: Derivation of a bronchial genomic classifier for lung cancer in a prospective study of patients undergoing diagnostic bronchoscopy
Source: BMC Med Genomics. 2015 May 6;8:18. doi: 10.1186/s12920-015-0091-3 (PMC4434538; doi:10.1186/s12920-015-0091-3)
Supplement: Additional file 9: — ROC curve analysis of the training set cohort using the finalized gene expression classifier. [file 12920_2015_91_MOESM9_ESM.docx]

**Additional file 9:** ROC curve analysis of the training set cohort using the finalized gene expression classifier


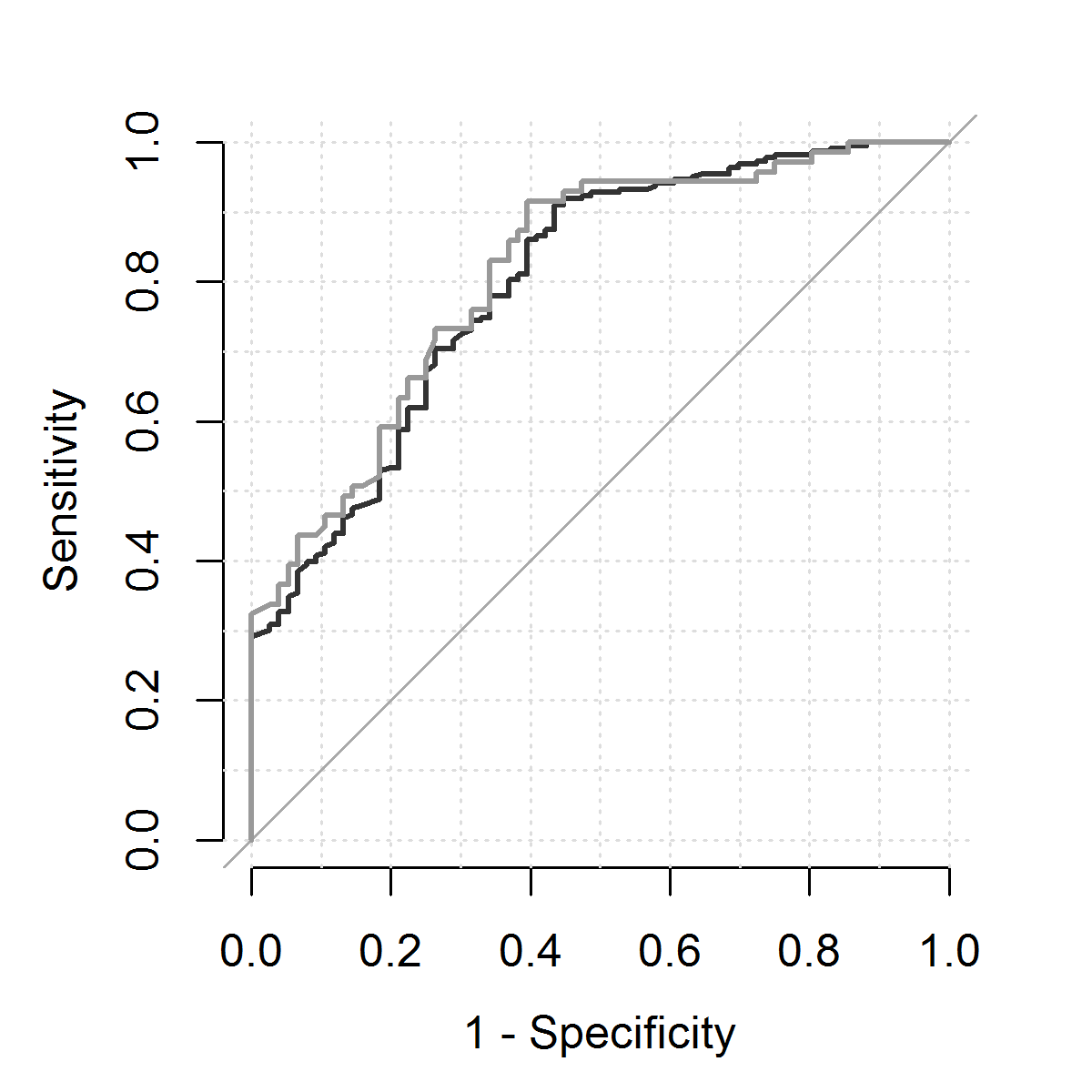


Gene expression data corresponding to all patients in the training set (black line), and the subset of patients with a non-diagnostic bronchoscopy (grey line) were analyzed using the locked classifier. The AUC was calculated as 0.78 (95% CI, 0.73-0.82) and 0.78 (95% CI, 0.71-0.85), for the two groups respectively.
