# Supplementary material for: Derivation of a bronchial genomic classifier for lung cancer in a prospective study of patients undergoing diagnostic bronchoscopy
Source: BMC Med Genomics. 2015 May 6;8:18. doi: 10.1186/s12920-015-0091-3 (PMC4434538; doi:10.1186/s12920-015-0091-3)
Supplement: Additional file 10: — Analysis of clinical characteristics and classifier performance according to race. [file 12920_2015_91_MOESM10_ESM.docx]

**Additional file 10:** Analysis of clinical characteristics and classifier performance according to race

|  | Caucasian | African-Am | *p* |
| --- | --- | --- | --- |
| N | 227 | 60 |  |
| Age | 62.7 | 63.5 | 0.67 |
| PY | 46.1 | 37.6 | 0.04 |
| Prevalence | 74% | 78% | 0.62 |
| AUC | 0.81 | 0.77 | 0.62 |
