# Supplementary material for: Derivation of a bronchial genomic classifier for lung cancer in a prospective study of patients undergoing diagnostic bronchoscopy
Source: BMC Med Genomics. 2015 May 6;8:18. doi: 10.1186/s12920-015-0091-3 (PMC4434538; doi:10.1186/s12920-015-0091-3)
Supplement: Additional file 11: — Biological characterization of classifier genes. [file 12920_2015_91_MOESM11_ESM.docx]

**Additional file 11:** Biological characterization of classifier genes.

| Cluster | Direction in Cancer | Biomarker genes | Biological themes |
| --- | --- | --- | --- |
| 1 | Down | BST1, CD177.1, CD177.2 | Innate immune response |
| 2 | Down | ATP12A, TSPAN2 | Mitotic cell cycle |
| 4 | Up | GABBR1, MCAM, NOVA1, SDC2 | Response to retinoic acid, cell cycle |
| 7 | Up | CGREF1, CDR1, CLDN22, NKX3-1 | Submucosal gland markers |
| 9 | Down | EPHX3, LYPD2 | Xenobiotic detoxification |
| 10 | Down | MIA, RNF150 | Cartilaginous markers |
